# Knockdown of endogenous RNF4 exacerbates ischaemia‐induced cardiomyocyte apoptosis in mice

**DOI:** 10.1111/jcmm.15363

**Published:** 2020-07-28

**Authors:** Fang Qiu, Yanna Han, Xiaoqi Shao, Petro Paulo, Wenyue Li, Mengying Zhu, Nannan Tang, Shuaili Guo, Yibing Chen, Han Wu, Dan Zhao, Yu Liu, Wenfeng Chu

**Affiliations:** ^1^ Department of Pharmacology (the State‐Province Key Laboratories of Biomedicine‐Pharmaceutics of China Key Laboratory of Cardiovascular Research Ministry of Education) College of Pharmacy Harbin Medical University Harbin P. R. China; ^2^ Center for Drug Research and Development Guangdong Pharmaceutical University Guangzhou P. R. China; ^3^ Departments of Clinical Pharmacy and Cardiology the 2nd Affiliated Hospital Harbin Medical University Key Laboratories of Education Ministry for Myocardial Ischemia Mechanism and Treatment Harbin P.R. China

**Keywords:** cardiomyocyte apoptosis, oxidative stress, p53, PML, reactive oxygen species, RNF4

## Abstract

RNF4, a poly‐SUMO‐specific E3 ubiquitin ligase, is associated with protein degradation, DNA damage repair and tumour progression. However, the effect of RNF4 in cardiomyocytes remains to be explored. Here, we identified the alteration of RNF4 from ischaemic hearts and oxidative stress‐induced apoptotic cardiomyocytes. Upon myocardial infarction (MI) or H_2_O_2_/ATO treatment, RNF4 increased rapidly and then decreased gradually. PML SUMOylation and PML nuclear body (PML‐NB) formation first enhanced and then degraded upon oxidative stress. Reactive oxygen species (ROS) inhibitor was able to attenuate the elevation of RNF4 expression and PML SUMOylation. PML overexpression and RNF4 knockdown by small interfering RNA (siRNA) enhanced PML SUMOylation, promoted p53 recruitment and activation and exacerbated H_2_O_2_/ATO‐induced cardiomyocyte apoptosis which could be partially reversed by knockdown of p53. In vivo, knockdown of endogenous RNF4 via in vivo adeno‐associated virus infection deteriorated post‐MI structure remodelling including more extensive interstitial fibrosis and severely fractured and disordered structure. Furthermore, knockdown of RNF4 worsened ischaemia‐induced cardiac dysfunction of MI models. Our results reveal a novel myocardial apoptosis regulation model that is composed of RNF4, PML and p53. The modulation of these proteins may provide a new approach to tackling cardiac ischaemia.

## 1 INTRODUCTION

Ischaemic heart disease which results from an imbalance between oxygen supply and demand is a leading cause of death worldwide. Myocardial ischaemia triggers catastrophic cardiomyocyte death resulting in systolic and metabolic dysfunction, and ultimately heart failure.^1^ Thus, understanding the mechanisms of ischaemic heart injury is a critical priority for cardiovascular researchers.

In the ischaemic myocardium, reactive oxygen species (ROS) are generated due to oxidative stimulation, such as lack of blood flow, serum withdrawal and pressure overload.^2^ ROS is a crucial mediator of the pathophysiology of post‐ischaemic heart dysfunction. Accumulation of intracellular ROS induces cardiomyocyte apoptosis,^3^ and the development of infarction is correlated with the number of apoptotic cardiomyocytes at peri‐infarction areas.^4^ Transgenic mice with overexpression of superoxide dismutase (SOD), an antioxidant protein, had significantly smaller infarction size compared to that of controls, which highlighted the vital role of ROS in the regulation of post‐ischaemic cardiac injury.^5^

Human ring‐finger protein 4 (RNF4), an E3 ubiquitin ligase, targets poly‐SUMO‐modified proteins, leading to protein degradation via the ubiquitin‐mediated pathway.^6^ RNF4 has been implicated in the proteolytic control of the degradation of promyelocytic leukaemia protein (PML) and in the transcriptional activity of multiple transcription factors, such as poly‐(ADP‐ribose) polymerase 1 (PARP‐1), hypoxia‐inducible factors (HIFs) and PEA3.^7^, ^8^, ^9^, ^10^ Moreover, RNF4 plays a role in the response of mammalian cells to DNA damage. Cells with RNF4 elimination are hypersensitive to certain types of DNA damage, and RNF4 deficiency results in inefficient end resection.^11^, ^12^ Furthermore, RNF4 is linked to tumorigenesis since RNF4 has been shown to enhance cancer cell survival by regulating Wnt and Notch pathways.^13^ A high level of RNF4 mRNA is related to poor survival among patients with breast cancer, and its protein expression is elevated in 30% of human colon adenocarcinomas.^14^ However, the role of RNF4 in heart disease has not yet been investigated.

Here, we examined RNF4 expression following myocardial infarction (MI) or H_2_O_2_/arsenic trioxide (ATO) treatment in vitro and in vivo. Upon oxidative stress, both in vitro and in vivo experiments revealed alterations in RNF4 expression whereby RNF4 first increased and then gradually decreased. Due to the rapid enhancement of RNF4, we presumed that reduction of RNF4 could contribute to alleviating oxidative injury. Nevertheless, knockdown of endogenous RNF4 exacerbated oxidative stress‐induced cardiomyocyte apoptosis and ischaemia‐induced cardiac dysfunction, which was associated with enhanced PML nuclear body (NB) accumulation and p53 recruitment and activation.

## 2 MATERIALS AND METHODS

### 2.1 Animals

All animal experiments complied with the regulations established by the Institutional Animal Care and Use Committee of Harbin Medical University and the NIH guidelines (Guide for the Care and Use of Laboratory Animals). All animal studies were approved by the appropriate ethics committee and have therefore been performed in accordance with the ethical standards established in the 1964 Declaration of Helsinki and its later amendments. Male Kunming mice (20‐25 g) were provided by the Experimental Animal Center of Harbin Medical University (Grade II). Mice were fed a standard chow diet, were provided tap water ad libitum and were housed under a controlled temperature (22°C) with a 12‐h light–dark cycle.

### 2.2 In vivo adeno‐associated virus infection

Mice were randomized into several groups (three groups: Sham, Scramble and shRNF4 groups; or four groups: Sham, MI, +Scramble and +shRNF4 groups). In vivo adeno‐associated virus infection was performed as described previously.^15^ Briefly, mice were anesthetized with sodium pentobarbital (40 mg⋅kg^−1^, *i.p.*). The heart was fully exposed via blunt dissection of subcutaneous tissue and the fourth intercostal muscle. The ascending aortic artery and main pulmonary artery were clamped. Then, 200 μL saline (Sham mice) or AVV9‐Scramble (1 × 10^9^ pfu at a volume of 200 μL, Scramble mice) or AVV9‐shRNF4 (1 × 10^9^ pfu at a volume of 200 μL, shRNF4 mice) was injected into the left ventricular cavity through the apex with a 30‐gauge syringe. After injection, the arteries remained occluded for 10 seconds. The shRNF4 sequence is listed in Table 1.

**Table 1:** Sequences of the specific siRNAs and shRNA, and the plasmids

Name	Sense (5’‐3’)	Antisense (3’‐5’)
siRNF4	GGAAACUGUUGGAGAUGAA	UUCAUCUCCAACAGUUUCC
sip53	ACCACUUGAUGGAGAGUAU	
shRNF4	GatccGGAAACTGTTGGAGATGAATTCAA GAGATTCATCTCCAACAGTTTCCTTTTTTg	AattcAAAAAAGGAAACTGTTGGAGATGA ATCTCTTGAATTCATCTCCAACAGTTTCCg
PML	TACCGGACTCAGATCTCGAGAT GGAAACTGAACCAGTTTCCGTG	GATCCCGGGCCCGCGGTACCGT GGCCAGGCATCCCTTACTTTCAG

Note

All the genes correspond to *Mus musculus*.

### 2.3 Mouse models of MI

Mice in the four groups were anaesthetized with sodium pentobarbital and then underwent left anterior descending coronary artery occlusion under sterile conditions. For Sham mice, a suture was crossed through the myocardium around the left anterior descending artery without ligation. Twenty‐four hours after MI, mice were subjected to heart function assessment and were then euthanized by cervical dislocation following CO_2_ inhalation. Heart tissue was harvested for subsequent experiments.

### 2.4 Isolation of neonatal mouse cardiomyocytes

Neonatal Kunming mice (1‐3 days old) were anaesthetized with 4% isoflurane inhalation before rapid heart excision. Hearts were dissected and cut into small pieces, as described previously.^16^ Briefly, cardiac tissue was digested with 0.25% trypsin. Isolated cells were cultured with fresh Dulbecco's modified Eagle's medium (DMEM, HyClone, Logan, UT, USA) with 10% foetal bovine serum (FBS, HyClone) at 37°C in 5% CO_2_, 95% air. After incubation for 1‐2 h, the media containing neonatal mouse cardiomyocytes (NMCMs) were removed into new 6‐well or 96‐well plates with the addition of 1% 5‐bromo‐2‐deoxyuridine (BrdU).

### 2.5 Drug administration and transfection

Cells were pre‐treated with Tempol (3 mM, MCE, USA) for 1 hours, which was then replaced with fresh DMEM (HyClone) without FBS (HyClone). Subsequently, H_2_O_2_ (200 μM, Sigma‐Aldrich, USA) or ATO (2 μM, Harbin YI‐DA Pharmaceutical Company, China) was added.

Small‐interfering RNAs (siRNAs) targeting RNF4 (siRNF4) or p53 (sip53), as well as a negative control (NC), were purchased from GenePharma (Shanghai, China). Cells were starved for 6 hours. The sequences of siRNF4 and sip53 are listed in Table 1. Next, siRNA and lipofectamine 2000 (Invitrogen, USA) were separately diluted with 250 μL of Opti‐MEM (Gibco, Grand Island, NY) for 5 minutes. Lipo and siRNA were then mixed. After 20 minutes, the mixture was added to the cells and cultured at 37°C for 6 hours. After incubation, cells were cultured with DMEM containing 10% FBS at 37°C.

Plasmids expressing mouse PML were purchased from GeneChem Co., Ltd, Shanghai, China. The sequence of PML is listed in Table 1. NMCMs were starved for 6 hours. The plasmid and X‐treme GENE HP reagent (Roche, Basel, Switzerland) were separately diluted with 250 μL of Opti‐MEM (Gibco, Grand Island, NY) for 5 minutes. Then, X‐tremeGENE HP reagent and plasmid were mixed. After 20 minutes, the mixture was added to the cells and cultured at 37°C for 6 hours. After incubation, cells were cultured with fresh DMEM with 10% foetal bovine serum at 37°C until needed.

### 2.6 Western blot

Total protein was extracted from cells and tissue with the addition of 20 mM N‐ethylmaleimide (Sigma‐Aldrich), and protein was quantified with a BCA kit. Protein samples were separated on 9% SDS‐PAGE gels and transferred to nitrocellulose‐filter membranes. Then, membranes were probed with the following primary antibodies against RNF4 (1:500; Creative Diagnostics, USA), GAPDH (1:10000; ABclonal, USA), actin (1:1000; Proteintech, USA), ubiquitin (1:600; Santa Cruz Biotechnology, USA), PML (1:1000; MBL, Japan), SUMO‐1 (1:200; Santa Cruz Biotechnology, USA), SUMO‐2/3 (1:500; Abcam, USA), p53 (1:800; Abcam, USA) and p‐p53 (1:1000; Cell Signaling Technology, USA). Immunoblots were observed by a LI‐COR Imaging System (LI‐COR Biosciences, Lincoln, NE), and Odyssey software was used to analyse band intensities (area × OD), which were normalized to GAPDH/Actin. Results are reported as fold changes normalized to control values.

### 2.7 Real‐time PCR

Total RNA was extracted from heart tissue at the per‐infarction area by using Trizol reagent (Invitrogen, Carlsbad, CA) according to the manufacturer's protocol. Extracted RNA was measured on a Nanodrop (Thermo Fisher Scientific, USA). Then, mRNA was quantified using SYBR Green I on an ABI 7500 fast real‐time PCR system (Applied Biosystems, USA). GAPDH was used as a reference gene. All primers used are shown in Table 2.

**Table 2:** The primers used for real‐time PCR to detect the mRNAs of targeting genes

Primer	Sense (5’‐3’)	Antisense (3’‐5’)
RNF4	CTGGAGTCGGTACGTCCTTG	GGAGCCAACTAGATGTGCAG
GAPDH	AAGAAGGTGGTGAAGCAGGC	TCCACCACCCGGTTGTTGCGC

Note

All the genes correspond to *Mus musculus*.

### 2.8 Terminal deoxynucleotidyl transferase dUTP nick‐end labelling (TUNEL) assay

Cell apoptosis was measured with a terminal deoxynucleotidyl transferase dUTP nick‐end labelling (TUNEL) assay using an In Situ Cell Death Detection Kit (Roche, Mannheim, Germany) according to the manufacturer's protocols (Roche). Briefly, cells or tissues were fixed in 4% paraformaldehyde for 1 hours, blocked with methanol with 3% H_2_O_2_ for 10 minutes, permeabilized with 0.1% Triton X‐100 in 0.1% sodium citrate for 2 minutes and incubated with TUNEL reaction mixture for 1 hours at 37°C in the dark. Nuclei were stained with 6‐diamidino‐2‐phenylindole (1:50; Beyotime Biotechnology, Shanghai, China) for 10 minutes. TUNEL‐positive cells were detected by laser‐scanning confocal microscopy (FV300, Olympus).

### 2.9 Detection of intracellular ROS levels

ROS assays were carried out as previously described.^17^, ^18^ To measure intracellular ROS levels in cardiomyocytes, NMCMs were cultured on sterile glass in 6‐well plates. After each drug treatment, the intracellular ROS level was detected using dihydroethidium, which is a fluorescent probe for ROS (Beyotime Biotechnology, Shanghai, China), according to the manufacturer's protocols (Beyotime Biotechnology). Briefly, cells were incubated with dihydroethidium (10 μM) in the dark for 20 minutes at 37°C. Thereafter, cells were washed twice and then observed by a laser‐scanning confocal microscope (Olympus, Tokyo, Japan) with an excitation wavelength of 370 nm and an emission wavelength of 594 nm. ROS in the representative fluorescent images were shown as green staining.

### 2.10 Measurement of cell viability

NMCMs were seeded into a 96‐well microplate at 4000 cells per well in 200 µL of media. After a 24‐h drug treatment, cells were incubated with 10 µL MTT solution (0.5 mg mL^−1^; Sigma‐Aldrich) for 4 hours. Each well was supplied with 100 µL of dimethyl sulphoxide (DMSO), and the microplate was shaken for 10 minutes. A microplate spectrophotometer (Tecan, Mannedorf, Switzerland) was used to measure the absorbance at 490 nm.

### 2.11 Echocardiographic measurement and pulse‐wave Doppler

An ultrasound machine (Vivid 7 GE Medical, General Electric Company, Fairfield, CT) with a 10‐MHz phase‐array transducer was used to assess ventricular function. Two‐dimensional and M‐mode images were obtained in the short‐axis view to assess systolic function. The left ventricular ejection fraction (EF) and fraction shortening (FS) were then calculated.

### 2.12 Immunofluorescent staining

NMCMs were cultured on a sterile glass in 6‐well plates. After drug treatment, samples were fixed in 4% paraformaldehyde for 15 minutes, permeabilized with Triton X‐100 (Beyotime, China), blocked in goat serum for 1 hour and then incubated with antibodies against PML (1:200; MBL, Japan) and p53 (1:800; Abcam, USA). DyLight 594‐conjugated goat anti‐chicken and goat anti‐mouse conjugated to fluorescein isothiocyanate (FITC; 1:500; Life Technologies) were used as secondary antibodies. After nuclear staining, a laser‐scanning confocal microscope (Olympus, Tokyo, Japan) was used to obtain fluorescent images at 430, 488 and 594 nm.

### 2.13 Electron microscopy

Cell and tissue samples were fixed in 2.5% glutaraldehyde (pH 7.4) overnight at 4°C and were then immersed in 0.1 M cacodylate buffer with 1% osmium tetroxide for 1 hour. Samples were dehydrated with a concentration gradient of ethanol and then embedded in Epon medium and dissected into 60‐ to 70‐nm sections. After being stained with uranyl acetate and lead citrate, sections were observed with a JEOL 1200 electron microscope (JEOL Ltd., Tokyo, Japan).

### 2.14 Masson staining

Cardiac tissue at the peri‐infarction area was sectioned and immersed in 4% paraformaldehyde, embedded in paraffin for 24 hours at 4°C and was then stained with Masson's trichrome (Accustain HT15, Sigma‐Aldrich). Image analysis software (Image‐Pro Plus v4.0; Media Cybernetics, Bethesda, MD, USA) was used to assess the extent of interstitial fibrosis.

### 2.15 Evans blue‐TTC staining

The infarcted area and the ischaemic area were determined by Evans blue‐TTC staining. 1% Evans blue solution 2 mL (Sigma‐Aldrich, St. Louis, USA) was injected via the femoral vein. The entire hearts were washed with cold phosphate‐buffered saline (PBS) and stored at −80°C. Frozen hearts were sliced into four pieces of the same thickness. The slices were immersed for 30 minutes into 1% triphenyltetrazolium chloride (TTC, Beyotime Biotechnology, Shanghai, China) solution at 37°C and were observed with a stereomicroscope. Normal area was stained blue, ischaemic area was stained red, and the infarcted tissue remained unstained pale. Infarct area, area at risk (AAR) and left ventricle size (LV) were analysed on each slice by image analysis software. The average ratios of AAR/LV (%) and infarct area/AAR (%) from four slices were used to denote the degree of myocardial ischaemia and infarct.

### 2.16 Extraction of cytoplasmic and nuclear protein

Nuclear protein extracts were obtained using a protein separation kit (Invent Biotechnology, Inc) according to the manufacturer's instructions. Heart tissue was washed with cold PBS and was then centrifuged at 5000 rpms for 5 minutes. The tissue was mixed with an appropriate volume of cytoplasmic extraction reagent, incubated on ice for 5 minutes and was then vortexed for 5 seconds at the highest speed. The above protocol was repeated five times. Samples were centrifuged for 5 minutes at 5000 rpms, and the supernatant (the cytoplasmic extract) was immediately transferred to a clean tube. Then, the nuclear extraction reagent was added and the samples were vortexed at the highest setting for 15 seconds. The above protocol was repeated five times. After centrifugation at 13 500 rpms for 15 minutes, the nuclear extract was transferred to a clean tube.

### 2.17 Statistical Analysis

Statistical analysis was performed with SigmaPlot (Systat Software, Inc, San Jose, CA, USA). Comparisons between two groups were determined by Student's *t* test. Multiple‐group comparisons were performed by one‐way ANOVA followed by Tukey's analysis for comparisons of mean values. *P* < .05 was considered statistically significant. All data are expressed as the mean ± standard deviation (SD).

## 3 RESULTS

### 3.1 Altered RNF4 expression upon oxidative stress in vivo and in vitro

**Figure 1:**
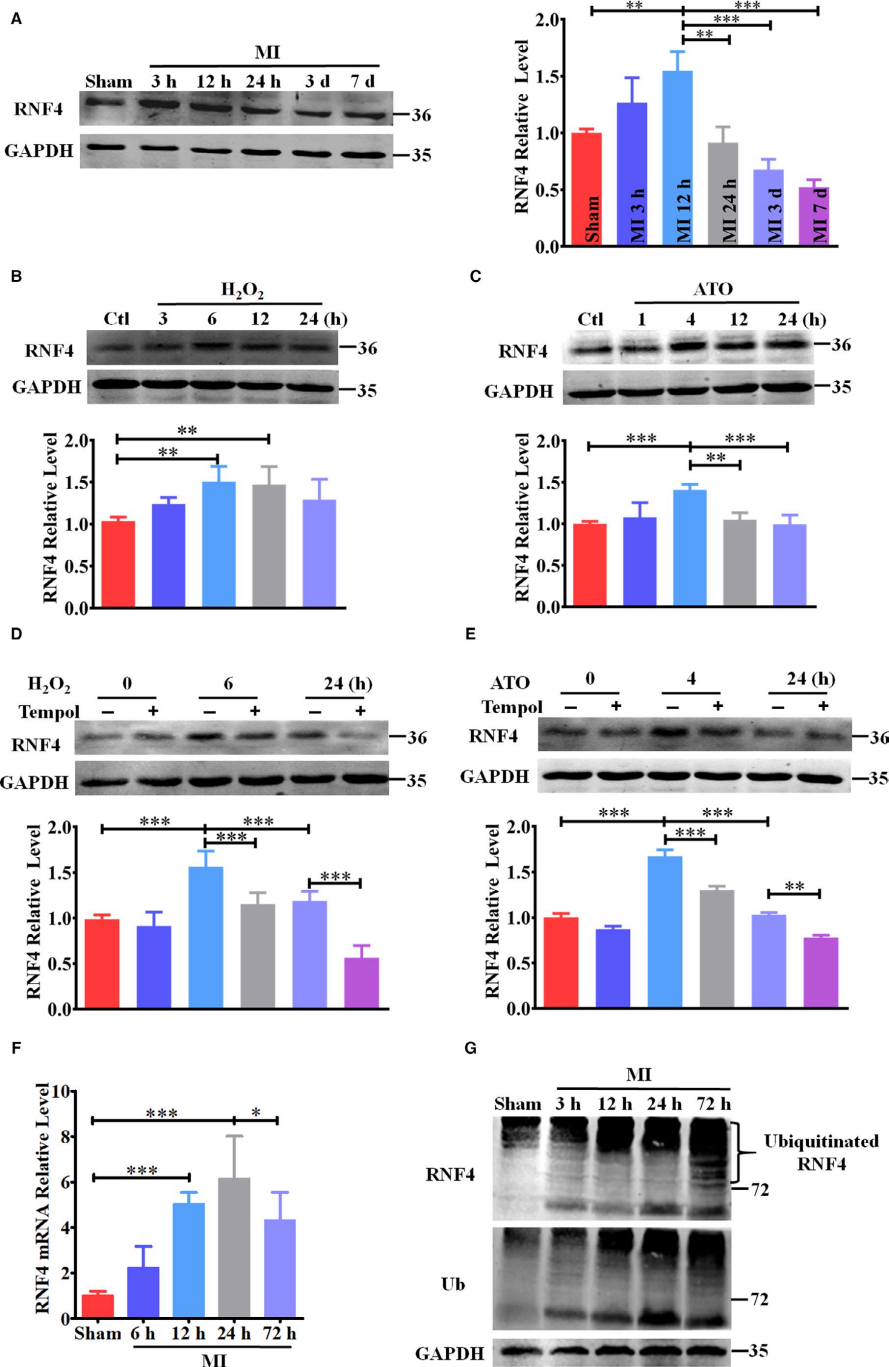
Altered pattern of RNF4 expression upon oxidative stress in vivo and in vitro. A RNF4 expression at peri‐infarction areas was detected by Western blot. n = 3. B RNF4 expression was examined by Western blot after H_2_O_2_ (200 μM) treatment in NMCMs. n = 5. C Western blot was performed after ATO (2 μM) treatment in NMCMs. n = 5. D and E After 1‐h pre‐treatment with Tempol (3 mM), RNF4 expression was measured by Western blot after H_2_O_2_ (D) or ATO (E) treatment in NMCMs. n = 3. F RNF4 mRNA levels at peri‐infarction areas were determined by real‐time PCR. n = 7. G Ubiquitin and RNF4 levels at peri‐infarction areas were detected by Western blot. Representative images from three independent experiments are shown. ^*^ *P* < 0.05, ^**^ *P* < 0.01, ^***^ *P* < 0.001

Mouse heart tissue from the peri‐infarction areas was harvested and then subjected to Western blot analysis to examine RNF4 expression under ischaemia‐induced oxidative stress. After MI, RNF4 increased dramatically and then decreased gradually at the peri‐infarction area (Figure 1A), but not at remote regions (Figure S1). H_2_O_2_ and ATO are often used as oxidants in vitro. As expected, RNF4 elevated initially and then attenuated after H_2_O_2_ (Figure 1B) or ATO (Figure 1C) treatment, while Tempol partially reversed the oxidative stimulus‐induced elevation of RNF4 in vitro (Figure 1D and E). To determine the mechanism of altered RNF4 expression upon oxidative stress, the transcription and degradation of RNF4 were examined in MI mice. RNF4 mRNA expression increased within 24 hours, followed by a decrease thereafter (Figure 1F). We also found that RNF4 degradation enhanced in a ubiquitin‐mediated pathway (Figure 1G). Hence, oxidative stress‐induced alteration in RNF4 expression was determined by both RNF4 transcription and degradation.

### 3.2 Knockdown of endogenous RNF4 exacerbates oxidative stress‐induced cardiomyocyte apoptosis in vitro

**Figure 2:**
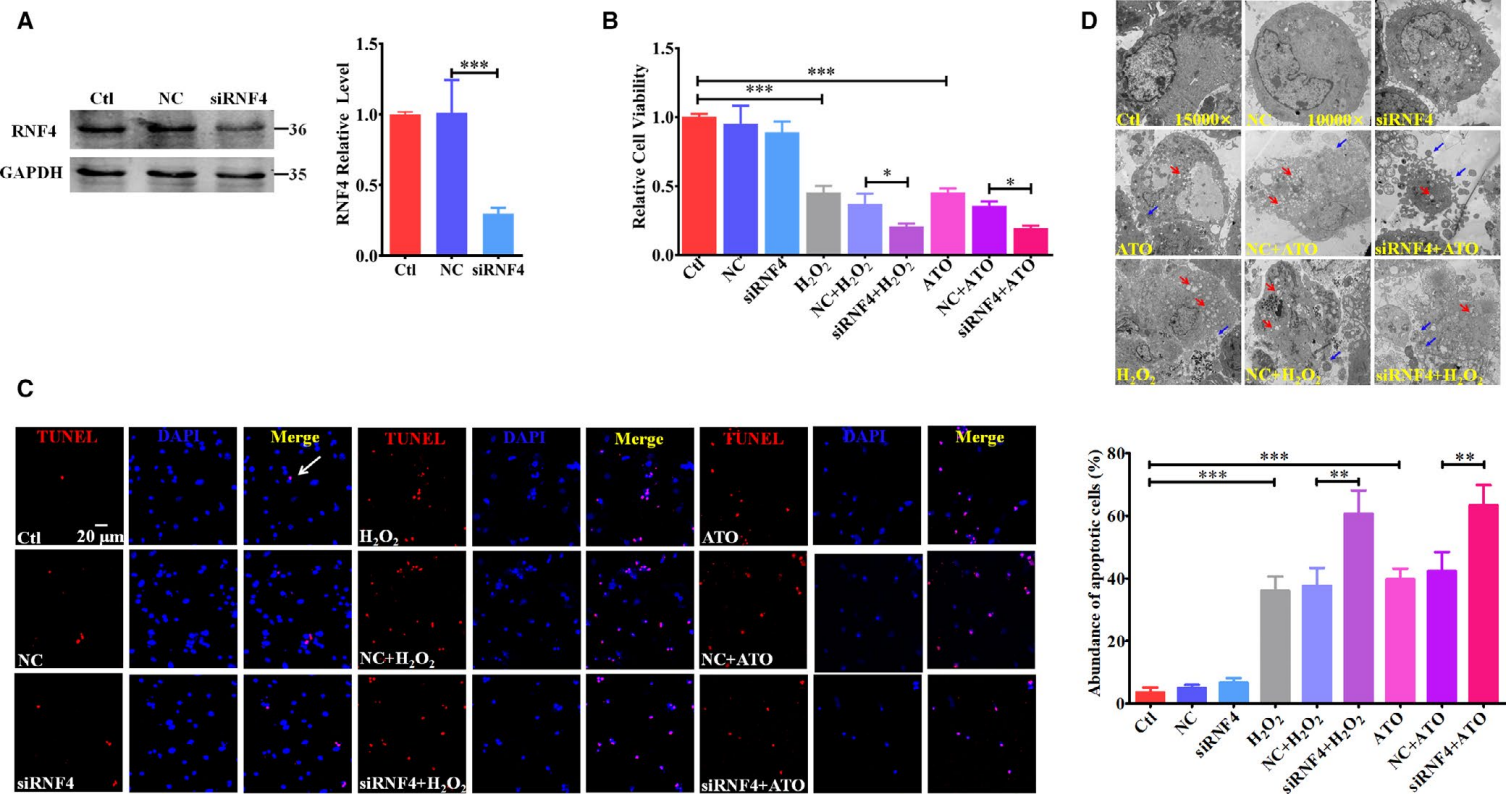
Knockdown of endogenous RNF4 exacerbates oxidative stress‐induced cardiomyocyte apoptosis in vitro. A RNF4 expression was examined by Western blot after siRNF4 transfection into NMCMs. B‐D NMCMs were treated with siRNF4 transfection and 24‐h H_2_O_2_ (200 μM)/ATO (2 μM) exposure. Cell viability was measured (B). Cell apoptosis was detected via TUNEL assays (C). The scale bar represents 20 μm. TUNEL‐positive cells are stained red, and nuclei are stained blue. Cell ultra‐structures were determined by EM (D). Red arrow: vacuolization of mitochondria. Blue arrow: apoptotic body. ^*^ *P* < 0.05, ^**^ *P* < 0.01, ^***^ *P* < 0.001. n = 3

Due to the rapidly increased levels of RNF4 upon oxidative stress, we next examined whether reduction of RNF4 could attenuate oxidative stress‐induced cellular injury. We transfected siRNF4 into NMCMs to knockdown endogenous RNF4 (Figure 2A). After 24‐h H_2_O_2_/ATO treatment, overall cell viability decreased by 55% compared to that of the control group (Figure 2B). Surprisingly, however, RNF4 knockdown reduced cardiomyocyte viability by 45% in the siRNF4 + H_2_O_2_ group compared to that of the NC + H_2_O_2_ group and by 46% in the siRNF4 + ATO group compared to that of the NC + ATO group (Figure 2B), suggesting that knockdown of endogenous RNF4 aggravated oxidative stress‐induced reduction of cell viability. The same results were obtained via TUNEL assays and electron microscopy (EM). The ratio of apoptotic cardiomyocytes increased significantly in the H_2_O_2_/ATO group compared with that of the control group (Figure 2C), and the ratio of TUNEL‐positive cells increased remarkably in the siRNF4 + H_2_O_2_/siRNF4 + ATO group compared with that of the NC + H_2_O_2_/NC + ATO group (Figure 2C). Distinct subcellular morphological changes, consistent with apoptosis, were found in terms of chromosomal condensation, apoptotic body formation and vacuolization of mitochondria after 24‐h H_2_O_2_/ATO treatment (Figure 2D). RNF4 knockdown induced more severe apoptotic phenomena, such as numerous apoptotic bodies, nuclei disappearance and cell structure corruption (Figure 2D). Moreover, intracellular ROS levels increased after 24‐h H_2_O_2_ treatment and RNF4 reduction enhanced oxidative stress‐induced ROS generation (Figure S2A), leading to further exacerbation of the oxidative injury to cardiomyocytes, while an ROS inhibitor significantly attenuated this injury (Figure S2B). These results indicate that even though oxidative stimuli increased RNF4 expression and triggered cardiomyocyte apoptosis, the reduction of endogenous RNF4 did not attenuate oxidative stress‐induced apoptosis, but rather, enhanced it.

### 3.3 Oxidative stress triggers an altered pattern of PML SUMOylation, and PML is involved in oxidative stress‐induced cell injury in vitro

**Figure 3:**
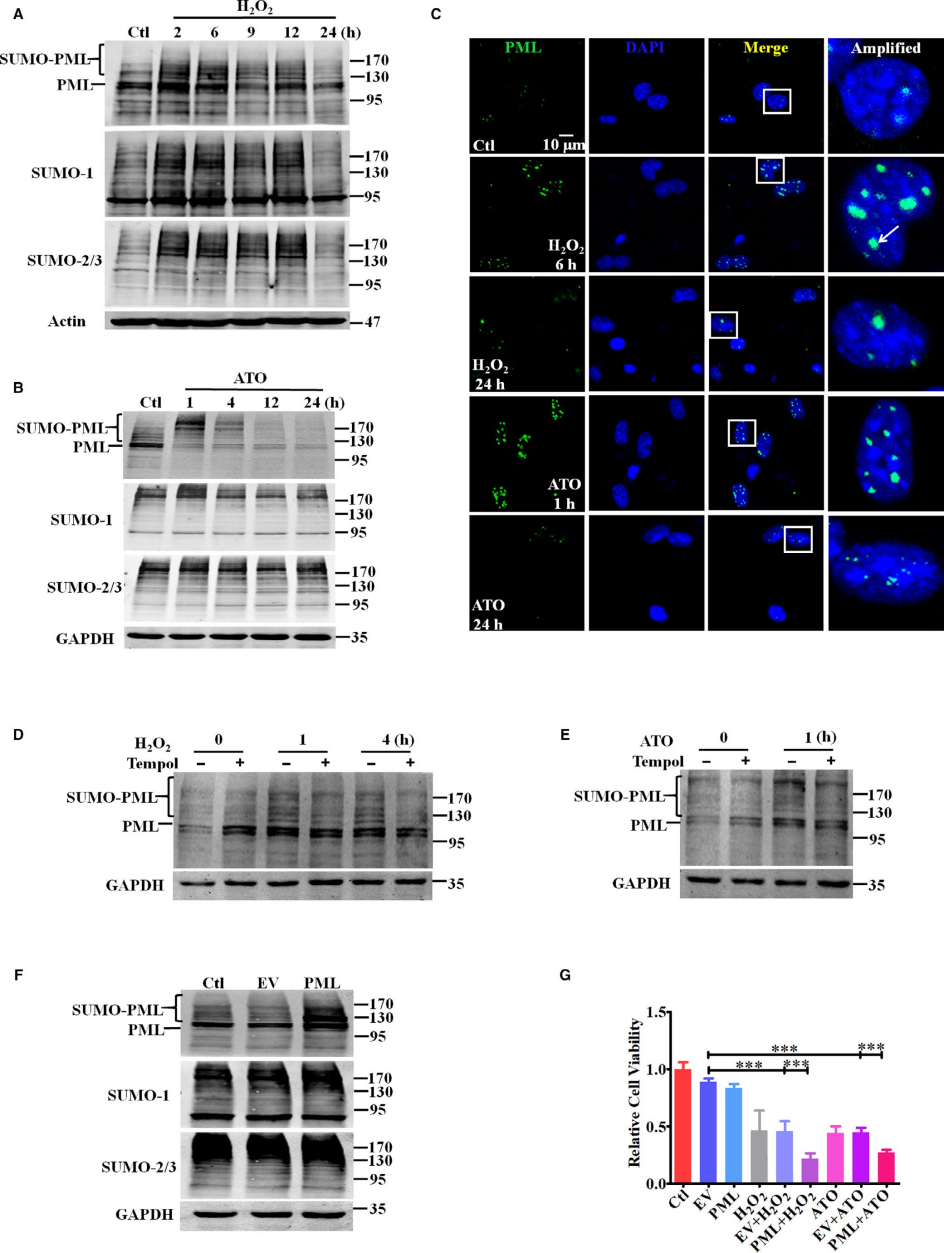
Oxidative stress triggers an altered pattern of PML SUMOylation, and PML is involved in oxidative stress‐induced cell injury in vitro. A PML, SUMO‐1 and SUMO‐2/3 expressions were detected via Western blot after H_2_O_2_ (200 μM) treatment. B NMCMs were treated with ATO (2 μM). Relevant protein expression was detected by Western blot. C Representative immunofluorescent images of PML (green) and DAPI (blue) are shown after H_2_O_2_/ATO treatment. The scale bar represents 10 μm. D and E After 1‐h pre‐treatment with Tempol (3 mM), PML expression was measured by Western blot after H_2_O_2_ (D) or ATO (E) treatment in NMCMs. F PML was overexpressed in NMCMs by plasmid transfection. Relevant protein expression was measured by Western blot. G NMCM viability was measured after PML overexpression and H_2_O_2_/ATO treatment for 24 hours. ^***^ *P* < 0.001. n = 3

PML is a ROS sensor.^19^ Upon oxidative stress, PML SUMOylation was initially enhanced and was then degraded thereafter (Figure 3A,B). The same result was recapitulated with immunofluorescent staining analysis. Compared that of the control group, the size and number of green‐stained PML‐NBs increased in nuclei after 6‐h H_2_O_2_ or 1‐h ATO treatment, while NBs were reduced to smaller sizes and numbers after treatment for 24 hours (Figure 3C), indicating a dynamic pattern of PML‐NBs during oxidative stimuli‐induced cardiomyocyte apoptosis. An ROS inhibitor partially reversed oxidative stress‐triggered PML SUMOylation (Figure 3D,E). PML was overexpressed in NMCMs (Figure 3F), and PML overexpression exacerbated H_2_O_2_/ATO‐induced cell injury (Figure 3G), indicating a role for PML in oxidative stimuli‐triggered downregulation of cell activity.

### 3.4 Knockdown of endogenous RNF4 attenuates oxidative stress‐induced PML‐NB degradation and enhances p53 recruitment and activation in vitro

**Figure 4:**
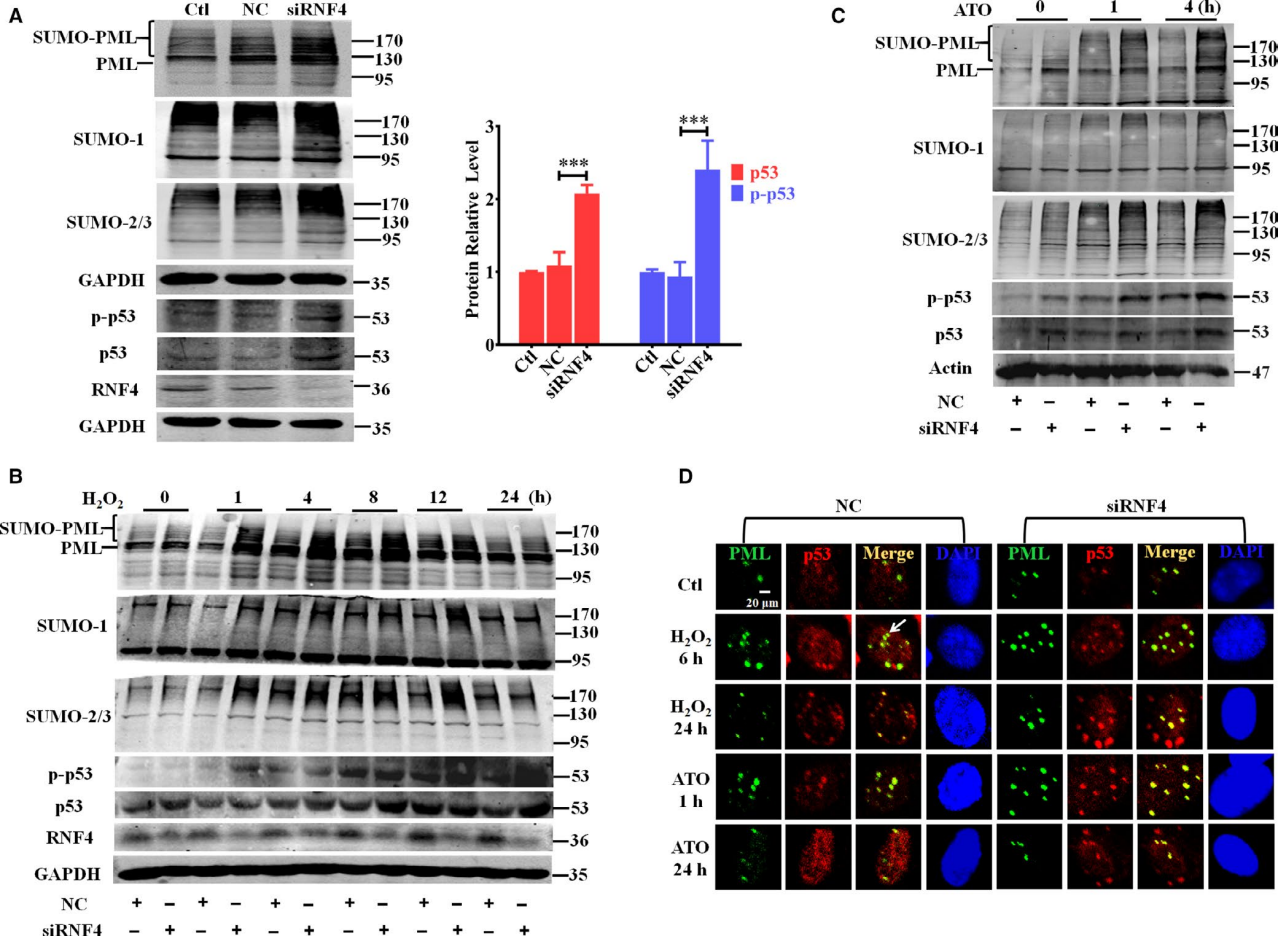
Knockdown of endogenous RNF4 attenuates oxidative stress‐induced PML‐NB degradation and enhances p53 recruitment and activation in vitro. A Protein expression of PML, SUMO‐1, SUMO‐2/3, p53, p‐p53 and RNF4 was measured by Western blot after siRNF4 transfection in NMCMs. B‐D After siRNF4 transfection, NMCMs were treated with H_2_O_2_ (200 μM) or ATO (2 μM). Relevant protein expression was examined by Western blot after H_2_O_2_ (B) or ATO (C) treatment. Representative immunofluorescent images of PML (green), p53 (red) and DAPI (blue) are shown. The scale bar represents 20 μm (D). ^***^ *P* < 0.001. n = 3

RNF4 can target the poly‐SUMO chain‐modified protein, leading to protein degradation via the ubiquitin‐dependent pathway. PML has been identified as the first and best‐characterized substrate of RNF4‐mediated degradation.^10^ Knockdown of RNF4 enhanced PML SUMOylation, which was accompanied by a twofold increase in the basal expression and activity of p53 compared with that of the NC group (Figure 4A). Meanwhile, knockdown of endogenous RNF4 not only enhanced oxidative stress‐induced PML SUMOylation and PML‐NB formation, but also attenuated oxidative stimuli‐induced PML and NB degradation (Figure 4B–D). Also, the reduction of RNF4 increased p53 expression and activation, as well as promoted p53 recruitment into NBs (Figure 4B–D). Thus, knockdown of RNF4 inhibited oxidative stimulation‐triggered PML degradation, which led to NB accumulation, promotion of p53 activation and co‐localization with NBs. These results suggest a potential role for RNF4 in cardiomyocyte apoptosis via regulation of PML and p53.

### 3.5 Knockdown of endogenous RNF4 deteriorates ischaemia‐induced cardiac dysfunction

**Figure 5:**
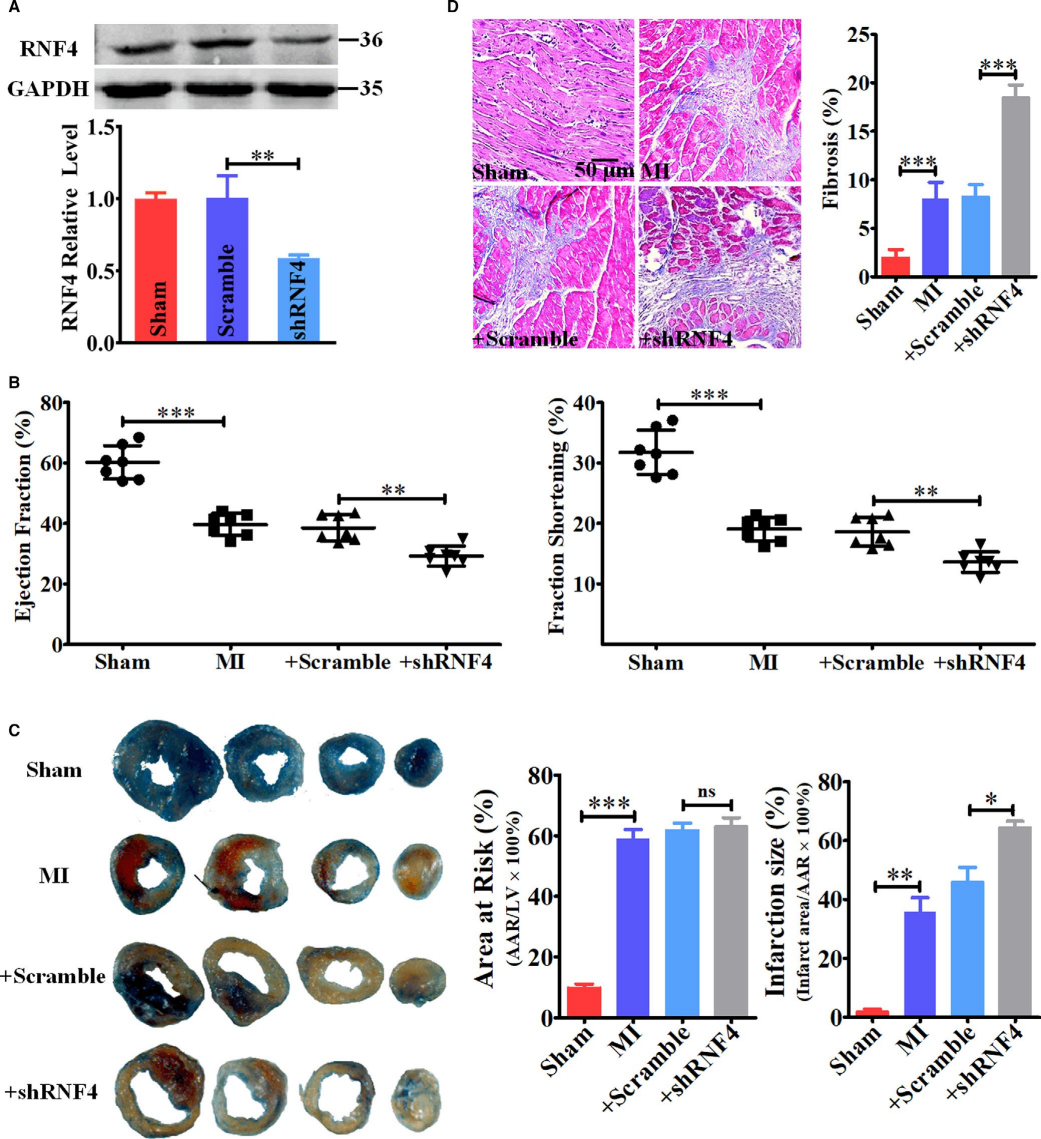
Knockdown of endogenous RNF4 exacerbates ischaemia‐induced cardiac dysfunction. A, Mice were treated with an in vivo infection of GFP‐AVV9‐shRNF4 for two weeks, and left ventricular tissue at the peri‐infarction area was harvested. RNF4 expression was measured. n = 3. B, After GFP‐AVV9‐shRNF4 infection for two weeks, mice underwent left anterior descending coronary artery occlusion for 24 hours. Cardiac function was evaluated by echocardiographic measurements. The ejection fraction and fraction shortening were calculated. n = 7. C, The infarcted area and the ischaemic area post‐24‐h MI was performed. Representative images from three independent experiments are shown. D, Representative Masson's staining images of the whole mouse heart after MI for 24 hours from three independent experiments are shown. The scale bar represents 50 μm. ^**^ *P* < 0.01. ^***^ *P* < 0.001

To investigate the role of RNF4 in heart disease, GFP‐labelled AAV9‐shRNA targeting RNF4 was delivered into the heart to specifically knockdown cardiac endogenous RNF4 via in vivo adeno‐associated virus infection, which is a well‐established experimental technique used in cardiovascular research.^20^, ^21^, ^22^ After two weeks, successful infection was demonstrated by green‐stained myocardia in the Scramble‐ and shRNF4‐treated groups (Figure S3), and endogenous RNF4 was reduced by 41% compared with that of the Scramble group (Figure 5A). Then, the effect of RNF4 knockdown on cardiac function after MI was examined. After infection for two weeks, mice were subjected to left anterior descending coronary artery occlusion for 24 hours, and cardiac function was then evaluated. A clear phenotypic manifestation was observed in mice of the four groups (Table 3). EF and FS of MI mice decreased by 34% and 40% compared to those of Sham mice, respectively (Figure 5B). Nevertheless, in accordance with the in vitro results, knockdown of RNF4 exacerbated ischaemia‐induced cardiac dysfunction, which was documented as reduced EF (by 24%) and FS (by 27%) in the + shRNF4 group compared with that of the + Scramble group (Figure 5B; Table 3). Then, the infarction size was measured and the peri‐infarction tissues underwent Masson's staining in 24‐h MI mice. A more extensive infarct and ischaemic area, as well as interstitial fibrosis, were shown in + shRNF4 mice compared to that in + Scramble mice (Figure 5C,D; Figure S4), indicating that RNF4 knockdown deteriorated post‐MI fibrotic remodelling. In order to determine the specific role of RNF4 in the heart, we detected the effect of RNF4 knockdown on cardiac function and structure in unstressed mice. Within 12‐week post‐shRNF4 infection, there were no significant changes in EF or FS of Sham and Scramble mice, while heart function of shRNF4 mice began to decline at eight‐week post‐infection (Figure S5A). In accordance with the results above, at twelve‐week post‐infection, extensive interstitial fibrosis and disrupted intercalated disks were present in shRNF4 hearts (Figure S5B), suggesting a specific effect of RNF4 on the heart.

**Table 3:** Echocardiography of mice infected with AVV9‐shRNF4 after 24‐h MI

	Sham (n = 7)	MI (n = 7)	+AVV9‐scramble (n = 7)	+AVV9‐shRNF4 (n = 7)
IVSd (mm)	0.89 ± 0.11	0.83 ± 0.08	0.84 ± 0.19	0.65 ± 0.11
IVSs (mm)	1.21 ± 0.22	1.04 ± 0.14	1.11 ± 0.22	0.74 ± 0.10^#^
LVIDd (mm)	3.99 ± 0.45	4.20 ± 0.44***	4.39 ± 0.18	4.37 ± 0.22
LVIDs (mm)	2.73 ± 0.42	3.40 ± 0.39	3.58 ± 0.18	3.78 ± 0.22
LVPWd (mm)	0.83 ± 0.13	0.74 ± 0.06	0.71 ± 0.08	0.65 ± 0.09
LVPWs (mm)	1.20 ± 0.23	0.95 ± 0.13	0.94 ± 0.26	0.72 ± 0.11
EF (%)	60.21 ± 5.54	39.64 ± 3.67***	38.56 ± 4.28	29.23 ± 3.29^###^
FS (%)	31.75 ± 3.69	19.07 ± 1.97**	18.57 ± 2.37	13.58 ± 1.67^##^

Note

Abbreviations: EF, ejection fraction; FS, fractional shortening; IVSd, interventricular septal thickness at diastole; IVSs, interventricular septal thickness at systole; LVIDd, left ventricular internal dimension at diastole; LVIDs, left ventricular internal dimension at systole; LVPWd, left ventricular posterior wall thickness at diastole; LVPWs, left ventricular posterior wall thickness at systole; MI, myocardial infarction.

AVV9‐siUbc9, adeno‐associated virus 9 carrying siRNA Ubc9; AVV9‐scramble, adeno‐associated virus 9 carrying scramble RNA. All data are expressed as mean ± SD.

** *P* < 0.01 vs Sham.

*** *P* < 0.001 vs Sham.

^#^ *P* < 0.05 vs +AVV9‐Scramble.

^##^ *P* < 0.01 vs +AVV9‐Scramble.

^###^ *P* < 0.001 vs +AVV9‐Scramble.

### 3.6 Knockdown of endogenous RNF4 aggravates ischaemia‐induced cardiomyocyte apoptosis in vivo

**Figure 6:**
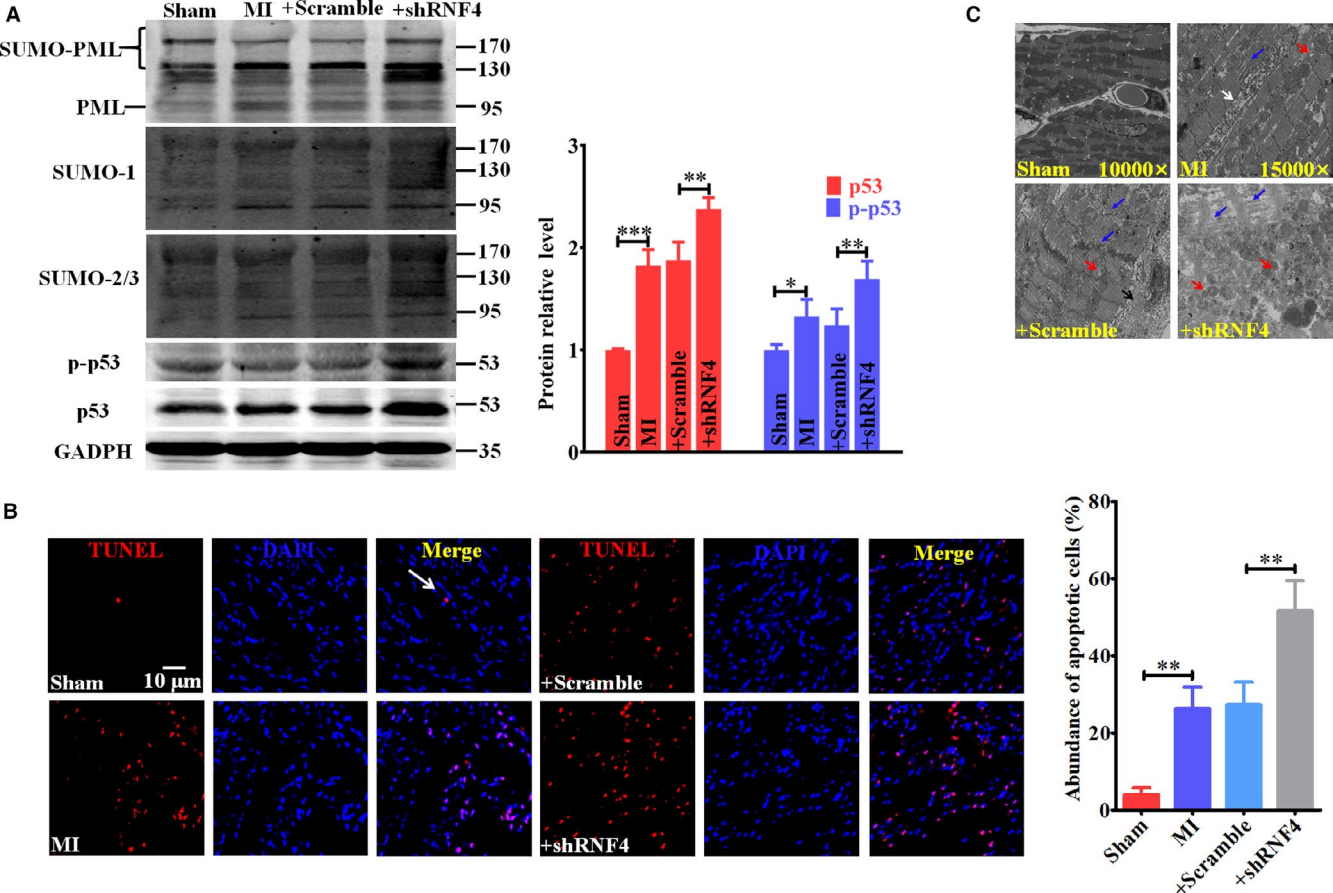
Knockdown of endogenous RNF4 aggravates ischaemia‐induced cardiomyocyte apoptosis in vivo. A‐C After infection of GFP‐AVV9‐shRNF4 for two weeks, mice underwent left anterior descending coronary artery occlusion for 24 hours. Relevant protein expressions of cardiac tissue at the peri‐infarction area were detected via Western blot (A). Representative images of TUNEL assays (B) and EM (C) are shown. The scale bar represents 10 μm. White arrow: pyknotic apoptotic nuclei. Red arrow: swollen mitochondria. Blue arrow: disrupted myofilaments and intercalated disks. Black arrow: interstitial collagen. ^*^ *P* < 0.05, ^**^ *P* < 0.01, ^***^ *P* < 0.001. n = 3

After 24‐h ischaemia in vivo, we detected increased PML SUMOylation and p53 expression and activity (Figure 6A) with pyknotic apoptotic nuclei (Figure 6C) at the peri‐infarction area. In accordance with the effect of RNF4 in vitro, specific knockdown of cardiac RNF4 promoted MI‐induced PML SUMOylation and p53 accumulation and activation (Figure 6A) due to the inhibitory effect of shRNF4 on PML degradation. Meanwhile, RNF4 knockdown‐enhanced p53 expression and activation led to an increasing number of apoptotic cardiomyocytes at peri‐infarction areas (Figure 6B), and the myocardium in the + shRNF4 group exhibited severely fractured and disordered structures with swollen mitochondria and disrupted myofilaments and intercalated disks compared to those in the + Scramble group (Figure 6C), which resulted in deterioration of cardiac dysfunction (Table 3; Figure 5B).

### 3.7 The PML/p53 axis is involved in the regulation of RNF4 knockdown‐induced deterioration of cardiomyocyte apoptosis

**Figure 7:**
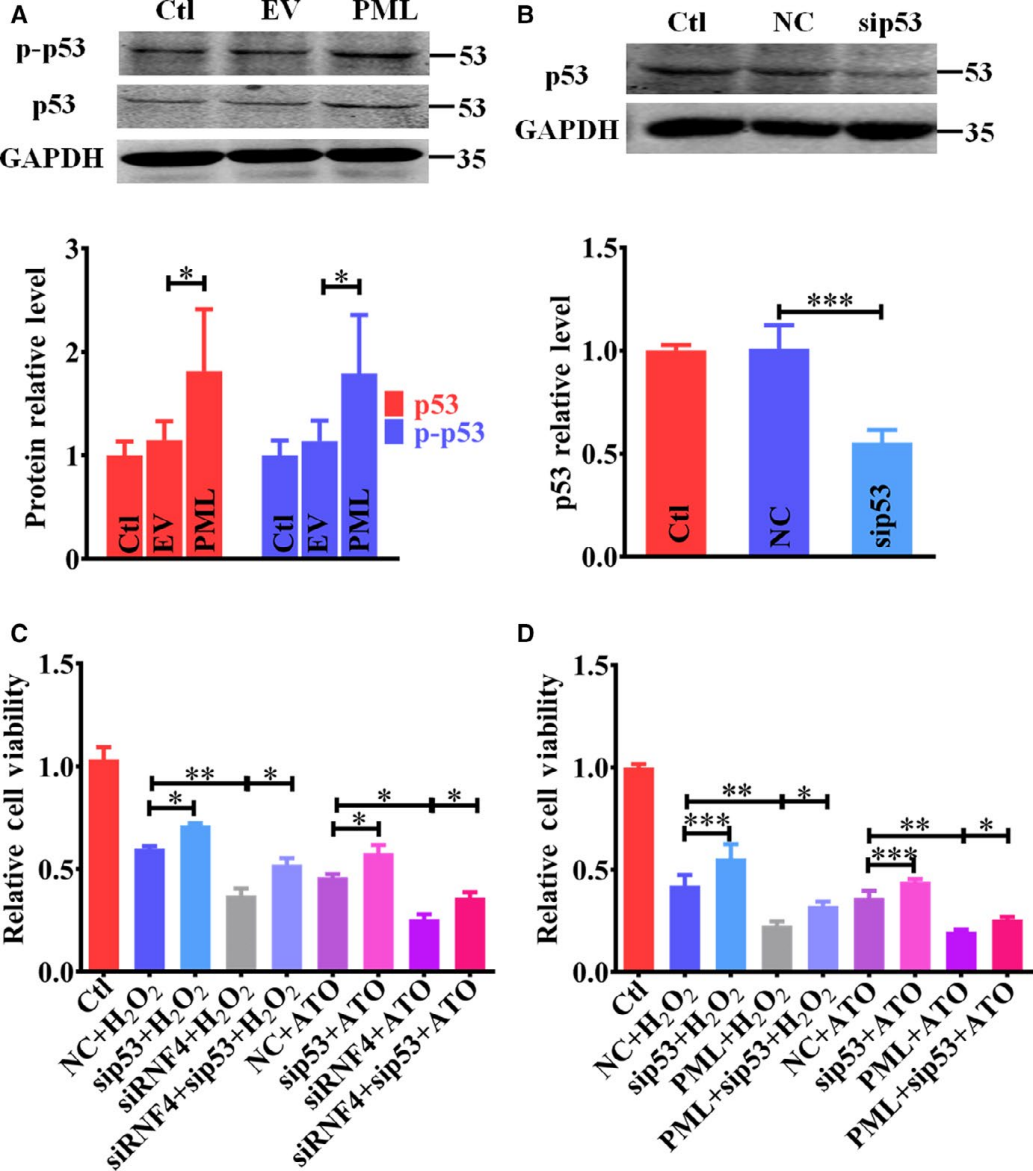
The PML/p53 axis is involved in the regulation of RNF4 knockdown‐induced exacerbation of cardiomyocyte apoptosis. A, p53 and p‐p53 were determined by Western blot after PML overexpression in NMCMs. B, p53 expression was examined by Western blot after sip53 transfection in NMCMs. C, NMCM viability was measured after sip53 transfection and H_2_O_2_ (200 μM)/ATO (2 μM) treatment for 24 hours. D, NMCM viability was measured after sip53 transfection and PML overexpression with H_2_O_2_/ATO treatment for 24 hours. ^*^ *P* < 0.05, ^**^ *P* < 0.01, ^***^ *P* < 0.001. n = 3

To further investigate the correlation between PML and p53 in regulating cell apoptosis, we overexpressed PML via transfecting plasmids carrying PML into NMCMs. PML is an important regulator of p53.^23^, ^24^ Overexpression of PML increased basal p53 expression and activity (Figure 7A). Knockdown of p53 by transfecting siRNA targeting p53 (Figure 7B) attenuated H_2_O_2_/ATO‐induced cell apoptosis and partially reversed the enhancement of RNF4 reduction and PML overexpression in oxidative stimuli‐triggered cell injury (Figure 7C,D). These results indicate a role for the PML/p53 axis in RNF4 knockdown‐induced exacerbation of cardiomyocyte apoptosis.

## 4 DISCUSSION AND CONCLUSIONS

**Figure 8:**
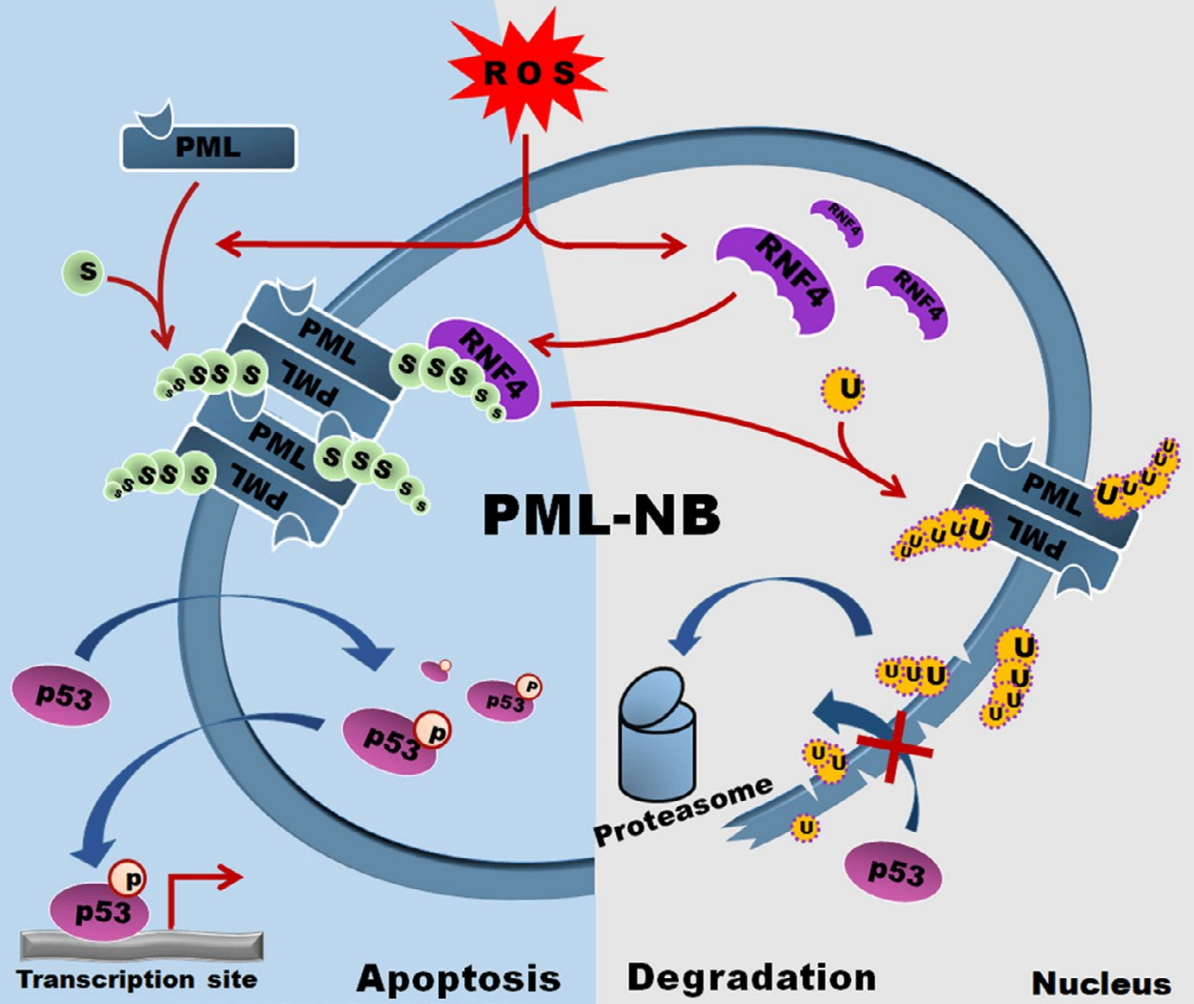
Graphical summary. Upon oxidative stress, ROS are generated. (1) ROS enhance PML SUMOylation and PML‐NB formation. Then, p53 is recruited into NBs and is stabilized by phosphorylation, which promotes its transcriptional activity, leading to p53‐dependent cell apoptosis; (2) ROS trigger a rapid increase in RNF4 expression, followed by a gradual decrease in RNF4 expression. RNF4 targets the SUMO chain of SUMOylated PML and modulates ubiquitin‐/proteasome‐mediated PML degradation, which breaks the pathway (1)

This is the first investigation to reveal an altered pattern of RNF4 upon exposure to oxidative stress in vivo and in vitro. However, in contrast to our original hypothesis, knockdown of RNF4 did not attenuate, but instead aggravated, cardiomyocyte apoptosis and heart injury. Upon oxidative stimulation, PML SUMOylation and PML‐NB formation were enhanced due to the elevated generation of ROS. Then, p53 was recruited into NBs and was stabilized by phosphorylation, which promoted its transcriptional activity and led to p53‐dependent apoptosis. However, ROS triggered a rapid increase in RNF4 expression that was followed by a gradual decrease in RNF4 expression. RNF4 targeted the SUMO chain of SUMOylated PML and modulated ubiquitin/proteasome‐mediated PML degradation, resulting in the abolishment of p53‐dependent apoptosis (Figure 8). Thus, knockdown of RNF4 inhibited PML degradation, which promoted NB accumulation and p53 activation and stabilization, resulting in oxidative injury.

ROS are generated during oxidative stress and are hallmarks of the pathophysiology of many diseases, including cancer, cardiovascular disease, neurodegeneration and ageing‐related disease.^25^, ^26^, ^27^ In all cases, ROS serve as important signalling modulators.^28^ During ischaemia, the large accumulation of ROS is the primary initiator of myocardial damage. ROS promote cardiomyocyte apoptosis, and the death of numerous cardiomyocytes leads to their replacement with collagen‐based scars.^2^ ROS can regulate p53 expression, DNA binding and transcriptional activity ^29^ and can also activate mitogen‐activated protein kinases (MAPKs) ^28^ and trigger intracellular calcium overload, leading to cell death.^30^ PML has been widely identified as an oxidation‐sensitive protein. A previous study showed that in vivo, ROS inducers, such as paraquat or paracetamol (acetaminophen), increase NB assembly very rapidly. In vitro, PML was also shown to dimerize through di‐sulphide bonds upon arsenic or H_2_O_2_, showing its sensitivity to oxidation. Oxidative stress promotes formation of NBs by enhancing PML multimerization through cysteine modifications in its RBCC domains, which may control PML expression and stability as well as its intracellular localization. NB‐associated substrate partners such as SUMO, SUMO enzymes like the UBC9 E2‐conjugating enzyme are then concentrated, and this subsequently increases PML and partner SUMOylation and increased PML NBs biogenesis.^26^, ^31^ And PML acts as a broad ROS sensor that regulates ROS homeostasis at least in part by enhancing p53 responses. Several studies by Lallemand‐Breitenbach et al have demonstrated that oxidative stress‐driven PML NBs formation regulates p53 post‐translational modification, triggering expression of a subset of p53 target genes, notably those mediating acute toxic response to ROS.^32^ Niwa‐Kawakita et al showed PML has a dual role upon oxidative stress, enforcing basal protection against ROS and facilitating ROS‐triggered apoptosis due to the multiple feedback loops and cross talk exist among ROS, PML and p53.^19^ This was consistent with our study that high ROS levels dramatically induced PML SUMOylation and NB aggregation, which was followed by a reduction of SUMOylated PML and NB due to long‐term oxidative stress‐triggered PML degradation (Figure 3). In our previous study, high‐molecular‐weight SUMOylated PML increased within two–three weeks after transverse aortic constriction (TAC) and then decreased after the eighth week,^21^ suggesting a dynamic pattern of PML SUMOylation upon stress. The dynamic process may be regulated by the multiple feedback loops and cross talk exist among the functional diversity of NB partner proteins.^26^, ^31^

Ubiquitination and SUMOylation are protein post‐translational modifications that have been strongly implicated in many cardiac pathophysiological processes, including ischaemic heart injury. Ubiquitination plays a critical role in removal of unwanted and no longer needed proteins in heart suffered ischaemic injury. Hu et al showed Ubqln1 exerts an essential role in coupling ubiquitination to proteasomal degradation in cardiomyocytes, and inadequate coupling between ubiquitination and proteasomal degradation as another major cause for the UPS functional deficit that thereby contributes to myocardial IRI.^33^ The modifications by SUMOylation affect the expression and function of proteins in ischaemic heart injury and attenuate or exacerbate the myocardial injury via different mechanisms. A previous research showed that SEARCA2a SUMOylation relieves Ca^2+^ overload in MI/R and reduces myocardial injury, SUMOylation promotes degradation of HDAC4 and reduces the production of ROS, eEF2 phosphorylated by Csk can be translocated to the nucleus by SUMOylation and induce apoptosis.^34^ Yi Gao et al reported that the nuclear receptor FXR is a SUMOylation target in heart tissues, and FXR SUMOylation levels are downregulated in response to MI/R injury. Consequently, the decreased SUMOylation augments the transcription activity of FXR, and the subsequently upregulates FXR target gene SHP mediated the detrimental effects of FXR in MI/R injury.^35^

Extensive research has revealed that RNF4, a conserved SUMO‐targeted ubiquitin E3 ligase, targets polySUMO‐modified proteins for ubiquitin‐mediated proteolysis. Lallemand‐Breitenbach et al demonstrated that arsenic‐induced PML SUMOylation triggers its polyubiquitination at Lys 48 and proteasome‐dependent degradation. When exposed to arsenic, SUMOylated PML recruits RNF4, as well as ubiquitin and proteasomes onto PML‐NBs.^36^ Tatham et al identified PML as the first protein degraded by SUMO‐dependent polyubiquitination. In the absence of RNF4, arsenic failed to induce degradation of PML and an accumulation of mixed, polyubiquitinated, poly‐SUMO chains as well as poly‐SUMOylated PML protein. As PML SUMOylation recruits not only RNF4, ubiquitin and proteasomes, but also many SUMOylated proteins with various physiological function onto PML nuclear bodies, these domains could physically integrate the SUMOylation, ubiquitination and degradation pathways and exhibited different, or even opposite regulatory functions.^37^ In other words, PML‐NBs have been associated with the regulation of several different cellular functions due to the functional diversity of NB partner proteins. RNF4 has also been reported to be implicated in proteolytic control, DNA repair and carcinogenesis; in the present study, we focused on its role in heart disease. An altered pattern of RNF4 expression was shown in the present investigation. After ischaemia, RNF4 increased significantly and was then gradually abated (Figure 1A‐C). It has been reported that RNF4 expression changes in a cell cycle‐dependent manner to regulate DNA repair. RNF4 accumulates in the S‐/G2‐phases but decreases in the G0/G1‐phases during DNA double‐strand break repair.^38^ Additionally, we found that MI induced more nuclear RNF4, but less cytoplasmic RNF4, which indicated that MI might trigger RNF4 trans‐localization (Figure S7). Phosphorylation has been reported to be the essential post‐translational modification of RNF4, which is required for RNF4‐mediated degradation of target proteins during DNA repair.^39^ Due to the oxidative stress‐induced would attenuate cardiac injury. Nevertheless, we found more severe cardiac dysfunction and cardiomyocyte apoptosis following MI. The underlying mechanism responsible for this effect is that knockdown of RNF4 inhibits PML degradation, resulting in p53 recruitment into PML‐NBs and p53‐dependent apoptosis, which aggravates ischaemic injury. We previously reported that manipulating PML SUMOylation could regulate cardiac fibrosis via silencing UBC9 and RNF4.^20^ In accordance with our present results, knockdown of RNF4 also aggravated cardiac fibrosis and dysfunction induced by TAC. As PML‐NB is a platform for the recruitment and interaction of multiple SUMOylated nuclear proteins, it is likely that other NB partner proteins are also potential targets of RNF4. Komaravelli et al found that respiratory syncytial virus (RSV) induced NRF2 (nuclear factor erythroid 2‐related factor 2) degradation in a RNF4‐dependent manner. NRF2 localizes, in part, to PML‐NBs, can undergo SUMOylation. Poly‐SUMOylated NRF2 is polyubiquitylated by RNF4 and subsequently degraded by the proteasome in PML‐NB domains. Silencing RNF4 expression rescued NRF2 nuclear levels and transcriptional activity, which alleviated RSV‐induced cellular oxidative damage.^18^ However, we did not detect an effect of RNF4 overexpression on myocardial apoptosis or cardiac function. And whether RNF4 overexpression could partially reverse oxidative stress‐induced cardiac injury remains to be studied in future.

In summary, our results demonstrate an altered pattern of RNF4 expression upon oxidative stress in vivo and in vitro. We also reveal that RNF4 is crucial for the regulation of post‐ischaemic myocardial apoptosis via modulation of the PML/p53 axis. Our findings provide novel evidence for future therapeutic approaches to treating cardiac ischaemia.

## CONFLICT OF INTEREST

All of the contributors in this study declared no conflict of interest.

## AUTHOR CONTRIBUTIONS

YL and WFC designed the experiments and supervised the project; FQ, YNH and XQS were the primary experimenters and were responsible for the writing of the manuscript; PP, MYZ and WYL established the animal models and conducted the in vivo experiments; NNT, SLG, YBC and HW conducted the in vitro experiments; and DZ performed the statistical analysis.

## Supporting information

Fig S1

Fig S2

Fig S3

Fig S4

Fig S5

Fig S6

Fig S7

Fig S8

## Data Availability

The data that support the findings of this study are available from the corresponding author upon reasonable request.
