# Supplementary figures and images for: Knockdown of endogenous RNF4 exacerbates ischaemia‐induced cardiomyocyte apoptosis in mice
Source: J Cell Mol Med. 2020 Jul 28;24(17):9545–59. doi: 10.1111/jcmm.15363 (PMC7520334; doi:10.1111/jcmm.15363)

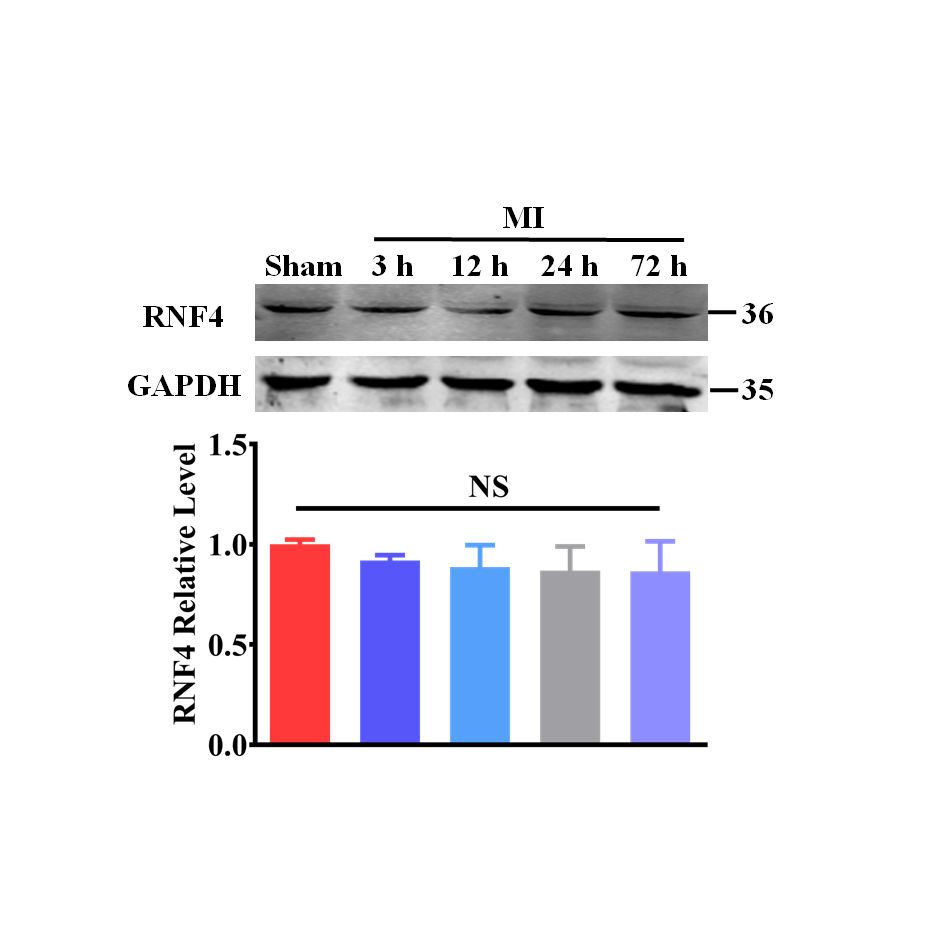

Supplement: Supplementary file 1 — Fig S1 [file JCMM-24-9545-s001.tif]

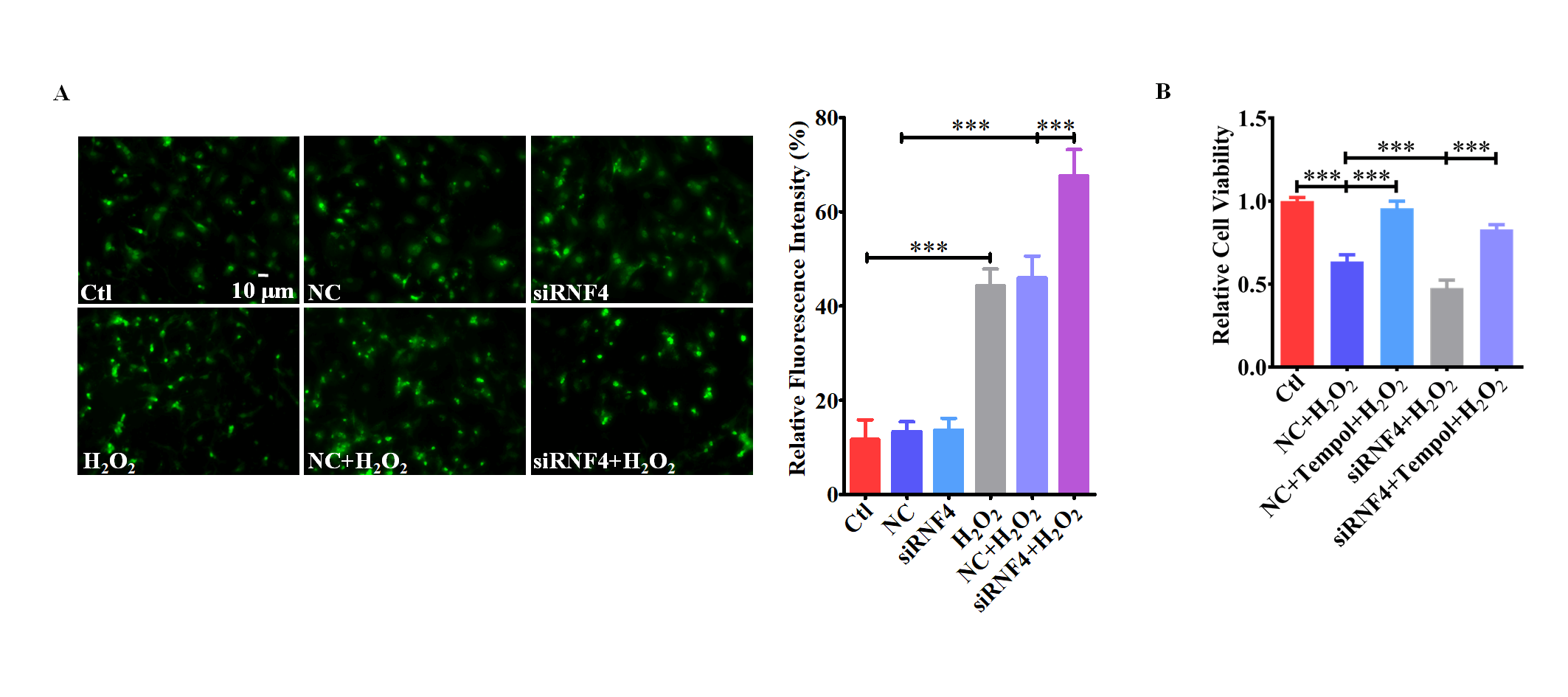

Supplement: Supplementary file 2 — Fig S2 [file JCMM-24-9545-s002.tif]

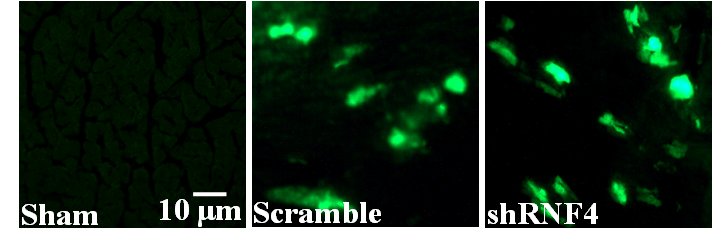

Supplement: Supplementary file 3 — Fig S3 [file JCMM-24-9545-s003.tif]

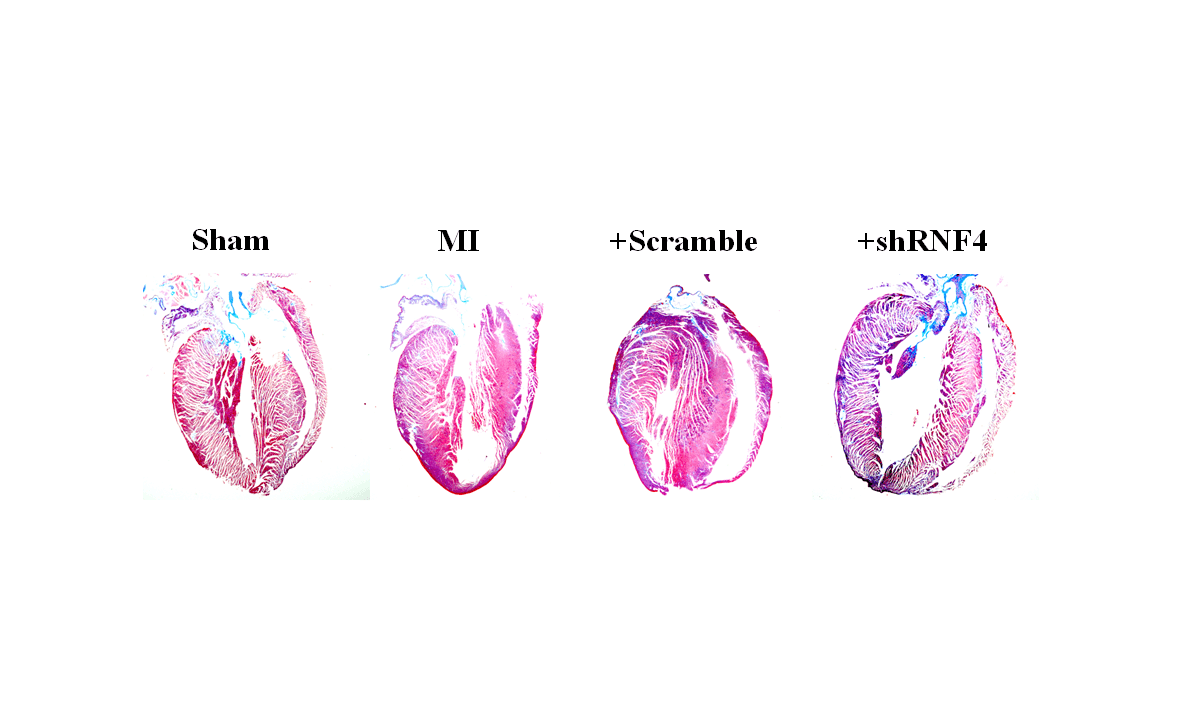

Supplement: Supplementary file 4 — Fig S4 [file JCMM-24-9545-s004.tif]

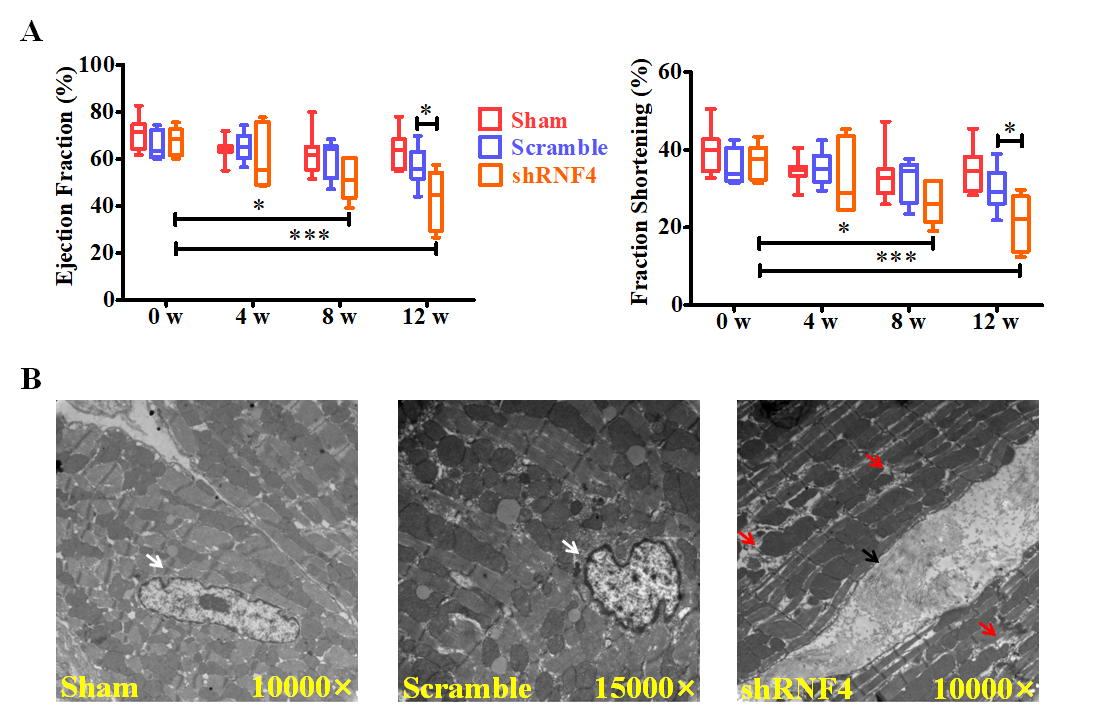

Supplement: Supplementary file 5 — Fig S5 [file JCMM-24-9545-s005.tif]

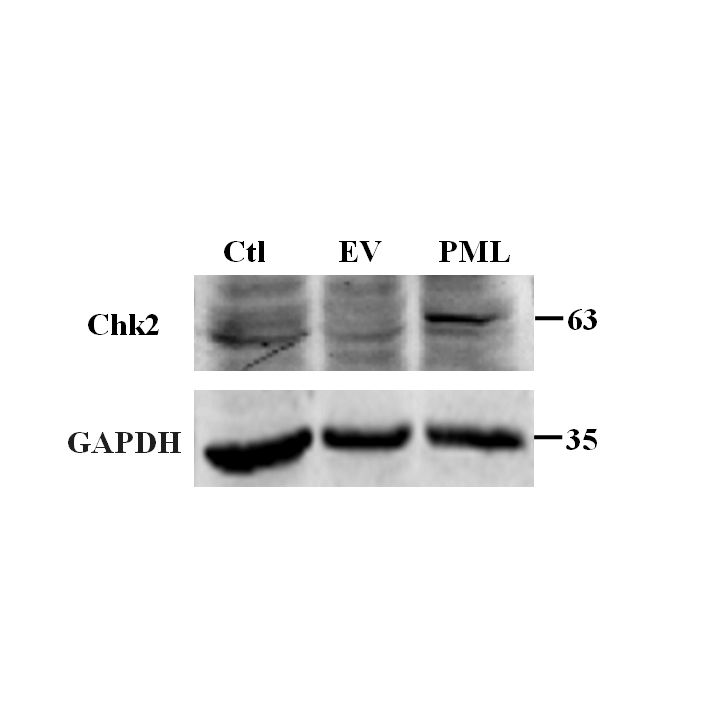

Supplement: Supplementary file 6 — Fig S6 [file JCMM-24-9545-s006.tif]

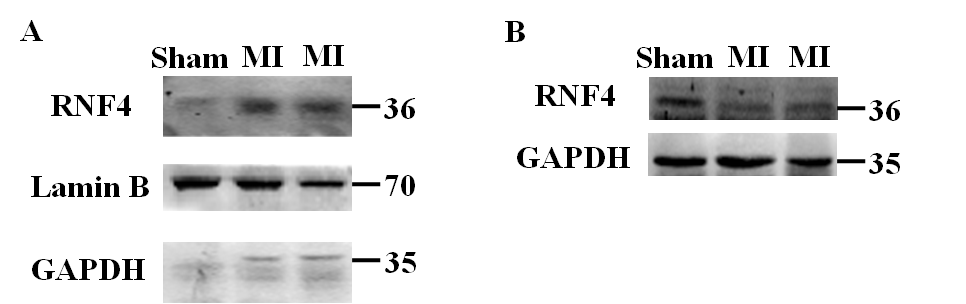

Supplement: Supplementary file 7 — Fig S7 [file JCMM-24-9545-s007.tif]

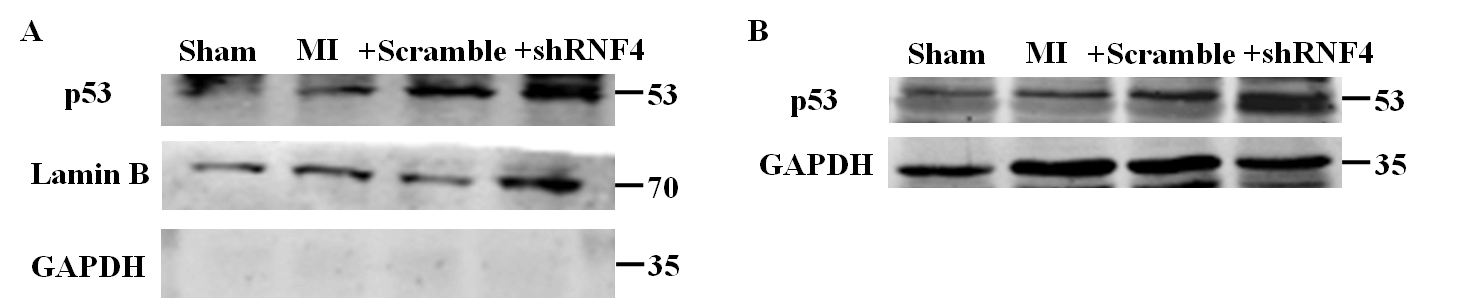

Supplement: Supplementary file 8 — Fig S8 [file JCMM-24-9545-s008.tif]
